# Socioeconomic inequalities across life and premature mortality from 1971 to 2016: findings from three British birth cohorts born in 1946, 1958 and 1970

**DOI:** 10.1136/jech-2020-214423

**Published:** 2020-10-06

**Authors:** Meg E Fluharty, Rebecca Hardy, George Ploubidis, Benedetta Pongiglione, David Bann

**Affiliations:** 1 UCL Institute of Education, Centre for Longitudinal Studies, London, UK; 2 UCL Institute of Education, Cohort and Longitudinal Studies Enhancement Resources, London, UK; 3 Bocconi University, Centre for Research on Health and Social Care Management, Milano, Italy

**Keywords:** Inequalities, Mortality, Socio-economic

## Abstract

**Introduction:**

Disadvantaged socioeconomic position (SEP) in early and adult life has been repeatedly associated with premature mortality. However, it is unclear whether these inequalities differ across time, nor if they are consistent across different SEP indicators.

**Methods:**

British birth cohorts born in 1946, 1958 and 1970 were used, and multiple SEP indicators in early and adult life were examined. Deaths were identified via national statistics or notifications. Cox proportional hazard models were used to estimate associations between ridit scored SEP indicators and all-cause mortality risk—from 26 to 43 years (n=40 784), 26 to 58 years (n=35 431) and 26 to 70 years (n=5353).

**Results:**

More disadvantaged SEP was associated with higher mortality risk—magnitudes of association were similar across cohort and each SEP indicator. For example, HRs (95% CI) from 26 to 43 years comparing lowest to highest paternal social class were 2.74 (1.02 to 7.32) in 1946c, 1.66 (1.03 to 2.69) in 1958c, and 1.94 (1.20 to 3.15) in 1970c. Paternal social class, adult social class and housing tenure were each independently associated with mortality risk.

**Conclusions:**

Socioeconomic circumstances in early and adult life show persisting associations with premature mortality from 1971 to 2016, reaffirming the need to address socioeconomic factors across life to reduce inequalities in survival to older age.

## INTRODUCTION

Previous evidence consistently shows disadvantaged socioeconomic position (SEP) in childhood and adult life is associated with increased premature mortality risk.^1^ However, the magnitude of the inequalities is likely context-specific and may therefore change across time. Evidence on these changes in the UK, however, is inconsistent. Inequalities in all-cause mortality by area-level measures of deprivation in adulthood appear to have increased from the 1980s to 2010s in Britain.^2^ This contrasts with reports of narrowing inequalities over the same period by educational attainment^3^—observed trends may therefore be sensitive to the specific SEP indicator used.

Existing studies investigating lifetime SEP and mortality associations have typically been limited to older cohorts (born in the 1930s–1950s), non-representative samples, and are limited to single indicators of SEP—with childhood indicators recalled in adulthood.^1^ The current study uses three comparable national British birth cohorts—born in 1946, 1958 and 1970—to investigate changes in inequalities in all-cause mortality risk across adulthood and early old age of three generations. The cohorts benefit from multiple prospectively ascertained SEP indicators. Previous evidence has examined the 1946 birth cohort in midlife^4^ and found childhood SEP was associated with premature mortality risk. Given persisting inequalities in multiple diseases and other mortality risk factors across the studied period (1971–2016),^4 5^ and the persisting inequalities in social and health outcomes in subsequent birth cohorts,^6 7^ we hypothesised that inequalities, according to both childhood and adult SEP, in premature mortality would have persisted.

## METHODS

### Study design and sample

We used data from three British birth cohort studies, which have reached mid-adulthood—born in 1946 (MRC National Survey of Health and Development (1946c)),^8 9^ 1958 (National Child Development Study (1958c))^10^ and 1970 (British Cohort Study (1970c)).^10^ These cohorts have been described in detail elsewhere.^6 7^ Analyses in the 1946c were weighted as this study consists of a social class-stratified sample. Participants were included in the current analysis if they were alive at age 26 years, had a valid measure of parental and/or own SEP and known vital status and date (from age 26 onwards).

Paternal occupational social class at birth was used in 1958c and 1970c and at age 4 in 1946c (birth data were not used to avoid World War II-related misclassification); occupation was classified using the Registrar General’s Social Class (RGSC) scale: I (professional), II (managerial and technical), IIIN (skilled non-manual), IIIM (skilled manual), IV (partly skilled) and V (unskilled) occupations. Maternal education collected at birth (1958–1970c) or age 6 (1946c) was distinguished using a binary variable indicating those who continued or left education at the mandatory leaving age.

At age 26 (1946c and 1970c) or 23 years (1958c), highest education was categorised into degree/higher, A levels/diploma, O levels/GCSEs or none; participant’s social class (RGSC) and housing tenure (owner vs renting; an indicator of wealth) were also collected.

### Mortality

Death notifications were supplied from the Office for National Statistics and/or via participants’ families during fieldwork.^11 12^

### Statistical analysis

To aid cross-cohort comparisons, analyses were carried out across the following age ranges: 26–43 years (all cohorts), 26–58 years (1946c–1958c) and 26–70 years (1946c).

For each SEP measure, cumulative death rates were calculated for each group. Cox proportional hazard models were used to estimate associations between each SEP indicator and all-cause mortality, following checks that the proportional hazard assumption held by calculating Schoenfeld residuals (online supplemental table S1). Follow-up was from age 26 to date of death—or was censored at date of emigration or at the end of each follow-up period for those still alive (age 43, 58 or 70). To provide single quantifications of inequalities, all SEP indicators were converted to ridit scores, resulting in an estimate of the Relative Index of Inequality. Cohort differences were formally tested using SEP×cohort interaction terms. Models were adjusted for sex, and also conducted separately to examine if findings differed in each sex. To investigate if associations of SEP across life and premature mortality were independent of each other and thus cumulative in nature, (1) mutually adjusted models were conducted including paternal and own social class—and additionally, housing tenure given the suggested importance of wealth^4^; (2) a composite lifetime SEP score was used in models, by combining these two or three indicators together and rescaling.^4^

10.1136/jech-2020-214423.supp1 Supplementary data

Multiple imputation was conducted to address missing data in SEP indicators (N=481 (1946c), N=514 (1958c), N=1236 (1970c)); complete case analyses yielded similar findings. Ten imputed data sets were used. Finally, to investigate if results were similar when examined in the absolute scale, models were repeated using logistic regression (dead/alive at the end of each follow-up period with those who emigrated excluded)—absolute differences in predicted probabilities of mortality were calculated. All analyses were conducted in Stata, version 16.0 (StataCorp LP, College Station, TX, USA).

## RESULTS

In total, 40 784 participants were included in analyses from 26 and 43 years (13% 1946c, 43% 1958c, 43% 1970c); 35 431 from 26 to 58 years (23% 1946c, 77% 1946c) and 5353 from 26 to 70 years (100% 1946c) (online supplemental tables S2 and S3). Mortality rates decreased sequentially across cohorts—by age 43, they were 74.53 per 1000 person years (1946c), 66.38 (1958c) and 51.63 (1970c); and by age 58, they were 116.94 per 1000 person years (1946c) and 103.66 (1958c).

**Figure 1:**
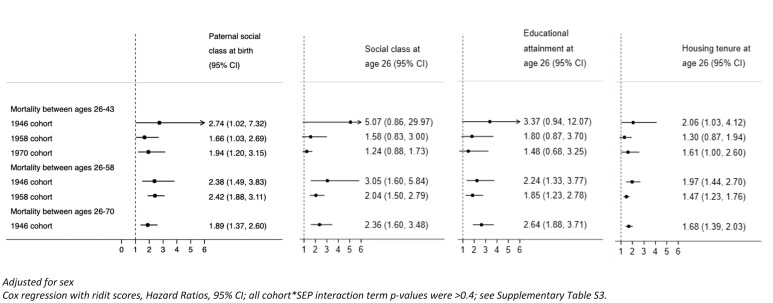
Associations between socioeconomic position and adult mortality risk: evidence from three British birth cohort studies.

More disadvantaged SEP in both childhood (paternal social class) and early adulthood (education attainment, own social class and housing tenure) was associated with higher mortality risk, with 21 of 24 hours (HRs) being between 1.6 and 3.1 (figure 1). As anticipated, associations were least precisely estimated at 43 years, where there were fewer deaths; and in the 1946c, which is smaller than 1970c and 1958c. Across each age period, HRs were generally larger in 1946c than the two later-born cohorts, but the CIs for 1946c at younger ages were wide (figure 1 and online supplemental table S3; all cohort×SEP interaction term p values were >0.4). For example, HRs of early death from 26 to 43 years comparing most to least disadvantaged paternal social class were 2.74 (95% CI 1.02 to 7.32) in 1946c, 1.66 (95% CI 1.03 to 2.69) in 1958c and 1.94 (95% CI 1.20 to 3.15) in 1970c. Associations were weaker for maternal education as an alternative indicator for childhood SEP, particularly for 1946c (online supplemental table S4). Housing tenure in adulthood was also associated with mortality, renters compared to homeowners had a consistently higher risk of death: HRs from 26 to 43 years were 2.06 (95% CI 1.03 to 4.12) in 1946c, 1.30 (95% CI 0.87 to 1.94) in 1958c and 1.61 (95% CI 1.00 to 2.60) in 1970c.

In models including both paternal and own social class, associations were typically partly attenuated, but generally both variables were still associated with premature mortality. Additionally, there was some evidence that composite lifetime SEP scores had larger magnitudes of association with mortality than each indicator in isolation (particularly in later periods of follow-up; online supplemental table S5a).

Findings were similar when housing tenure was included in models (online supplemental table S5b).

Findings of persistent inequalities in premature mortality across each cohort were also found when examining on the absolute scale (online supplemental tables S6), and when conducted separately among men and women (online supplemental tables S7 and S8). There was suggestive evidence for stronger associations among females in the 1946c and among males in the 1970c.

## DISCUSSION

Despite declining mortality rates across the studied period (1971–2016), inequalities in premature mortality appear to have persisted and were consistently found for multiple SEP indicators in early and adult life. Our findings build on prior investigations which used 1946c but not younger cohorts^4^ or repeated follow-up of adult cohorts^1 13^; and seminal reviews which focus on area-based SEP indicators.^2^ ^14^

The persistence of inequalities, even in a period of marked changes to cultural, social, economic and population-wide health (eg, declines in CVD mortality rates) is suggestive of multiple time-depending pathways between SEP and mortality.^15^ It is possible that, despite their overlap, each SEP indicator captures different pathways, resulting in their independent associations with mortality. For example, child SEP is associated with many mortality risk factors such as BMI independently of adult SEP,^6^ and housing tenure may specifically capture wealth given the increasing value of housing in Britain—wealth is increasingly suggested to be an important health-relevant SEP indicator.^16^ The main causes of death within these cohorts were likely to have been cancers, coronary heart disease and unnatural causes.^17^

Strengths of the study include the use of three large nationally representative studies, enabling long-run investigation mortality risk trends, and use of multiple SEP indicators across life. While we use multiple indicators of SEP, they are likely to be underestimates of socioeconomic inequality—wealth for example is only crudely approximated by home ownership, we lack comparable data on income and lacked power to investigate highest attained social class in midlife. Further, while RGSC is widely used in historic samples and official statistics (pre-2000), there is uncertainty in the criteria with which jobs were classified. While there were a small number of participants with missing outcome data, reassuringly the mortality rates in each cohort corresponded with the expected population at the time.^18^ Our study was limited to all-cause mortality; however, trends in inequalities may differ by health outcome, for example, absolute inequalities in coronary heart disease appear to have narrowed in 1994–2008,^19 20^ but inequalities in stroke remained unchanged.^20^ Future studies with larger sample sizes are warranted to investigate trends in cause-specific premature mortality.

Our findings reaffirm needs to address socioeconomic factors in both early and adult life to reduce inequalities in early-mid adulthood mortality. In contemporaneous and future cohorts, inequalities in premature mortality are likely to be significant barriers to a necessary component of healthy ageing: survival into older age.

**What is already known on this subject**

- Disadvantaged socioeconomic position in early and adult life is associated with increased premature mortality risk. Relative inequalities in all-cause mortality by area deprivation have increased from the 1980s to 2010s in England, Wales and Scotland. However, this contrasts with reports of narrowing relative mortality inequalities by educational attainment, and these differences in trends by SEP assessment suggest differences in social stratification according to different measures. Therefore, while there is a known association of SEP with mortality, there is little evidence on how different SEP indicators are associated with mortality risk, and how these associations have changed across time.

**What this study adds**

- Existing studies investigating lifetime SEP and mortality associations have typically been limited to older cohorts, non-representative samples, and are limited to single indicators of SEP—with childhood indicators recalled in adulthood. The current study uses three comparable national British birth cohorts—born in 1946, 1958 and 1970—to investigate changes in inequalities in all-cause mortality risk across adulthood and early old age of three generations. The cohorts benefit from multiple prospectively ascertained SEP indicators across life. Our study finds that despite declining mortality rates, inequalities in premature mortality appear to have persisted and were consistently found for multiple SEP indicators in early and adult life.
