# Supplementary material for: Socioeconomic inequalities across life and premature mortality from 1971 to 2016: findings from three British birth cohorts born in 1946, 1958 and 1970
Source: J Epidemiol Community Health. 2020 Oct 6;75(2):193–6. doi: 10.1136/jech-2020-214423 (PMC7815902; doi:10.1136/jech-2020-214423)
Supplement: Supplementary data [file jech-2020-214423supp001.pdf]

Supplementary Table S1. Results of proportional hazards assumption tests

		Mortality from 26-43		Mortality from 26-58		Mortality from 26-70	
		chi2	*p*	*chi2*	*p*	*chi2*	*p*
**BCS 1970**	Paternal SEP at birth	0.45	0.800				
	Maternal education at birth	3.19	0.203				
	Own SEP at age 26	0.67	0.717				
	Own education at age 26	1.77	0.414				
	Housing tenure at 26	0.44	0.804				
**NCDS 1958**	Paternal SEP at birth	4.69	0.096	6.50	0.039		
	Maternal education at birth	3.40	0.183	5.58	0.061		
	Own SEP at age 23	0.79	0.675	2.15	0.342		
	Own education at age 23	1.14	0.566	1.13	0.568		
	Housing tenure at 23	0.63	0.731	1.65	0.439		
**NSHD 1946**	Paternal SEP at birth	0.54	0.764	0.06	0.968	2.15	0.341
	Maternal education at age 6	2.18	0.337	2.49	0.288	4.30	0.116
	Own SEP at age 26	2.06	0.357	3.23	0.199	0.82	0.663
	Own education at age 26	0.48	0.786	0.25	0.884	0.99	0.611
	Housing tenure at 26	0.67	0.714	0.35	0.841	5.02	0.081

Adjusted for sex

Table S2. Socioeconomic indicators and mortality status in the 1946, 1958, and 1970 cohort

			Mortality from 26-43						Mortality from 26-58						Mortality from 26-70					
			Alive		Dead		.		Alive		Dead		.		Alive		Dead		.	
			N	*(%)*	*N*	*(%)*	*N*	*(%)*	*N*	*(%)*	*N*	*(%)*	*N*	*(%)*	*N*	*(%)*	*N*	*(%)*	*N*	*(%)*
**BCS 1970**	Paternal social class at birth	I Professional etc.	786	95.85	10	1.22	24	2.93												
		II Intermediate	1832	96.12	15	0.79	59	3.1												
		III Non-manual skilled	1834	95.32	23	1.2	67	3.48												
		III Manual skilled	7104	94.17	123	1.63	317	4.2												
		IV Partly skilled	2316	93.65	42	1.7	115	4.65												
		V Unskilled	1027	92.86	20	1.81	59	5.33												
		.	3185	95.93	45	1.36	90	2.71												
	Maternal Education at birth	Continued school	5510	95.25	69	1.19	206	3.56												
		Left school	10583	93.95	178	1.58	503	4.47												
		.	1991	97.41	31	1.52	22	1.08												
	Own social class at age 26	I Professional etc.	458	99.57	2	0.43	0	0												
		II Intermediate	2219	99.15	19	0.85	0	0												
		III Non-manual skilled	1813	99.07	17	0.93	0	0												
		III Manual skilled	1142	97.94	22	1.89	2	0.17												
		IV Partly skilled	906	98.59	13	1.41	0	0												
		V Unskilled	177	98.88	1	0.56	1	0.56												
		.	11369	92.42	204	1.66	728	5.92												
	Own highest education at age 26	Degree/higher	2088	98.96	21	1	1	0.05												
		A levels/ diploma	881	98.55	13	1.45	0	0												
		O levels / GCSE	4852	98.86	54	1.1	2	0.04												
		None	476	97.74	10	2.05	1	0.21												
		.	9787	91.52	180	1.68	727	6.8												
	Housing tenure at age 26	Own	3775	99.11	34	0.89	0	0												
		Rent/other	4427	98.38	69	1.53	4	0.09												
		.	9882	91.64	175	1.62	727	6.74												

**NCDS 1958**	Paternal social class at birth	I Professional etc.	711	95.31	6	0.8	29	3.89	701	93.97	16	2.14	29	3.89
		II Intermediate	2007	94.09	27	1.27	99	4.64	1949	91.37	85	3.98	99	4.64
		III Non-manual skilled	1487	93.4	24	1.51	81	5.09	1455	91.39	56	3.52	81	5.09
		III Manual skilled	7744	92.45	113	1.35	519	6.2	7418	88.56	439	5.24	519	6.2
		IV Partly skilled	1861	93.28	23	1.15	111	5.56	1777	89.07	107	5.36	111	5.56
		V Unskilled	1460	90.35	38	2.35	118	7.3	1364	84.41	134	8.29	118	7.3
		.	1988	94.67	25	1.19	87	4.14	1905	90.71	108	5.14	87	4.14
	Maternal Education at birth	Continued school	4085	94.19	46	1.06	206	4.75	3952	91.12	179	4.13	206	4.75
		Left school	12000	92.19	199	1.53	817	6.28	11489	88.27	710	5.45	817	6.28
		.	1173	97.34	11	0.91	21	1.74	1128	93.61	56	4.65	21	1.74
	Own social class at age 23	I Professional etc.	430	98.85	2	0.46	3	0.69	418	96.09	14	3.22	3	0.69
		II Intermediate	2095	98.08	29	1.36	12	0.56	2020	94.57	104	4.87	12	0.56
		III Non-manual skilled	3942	98.55	51	1.27	7	0.18	3825	95.63	168	4.2	7	0.18
		III Manual skilled	2956	98.01	46	1.53	14	0.46	2837	94.06	165	5.47	14	0.46
		IV Partly skilled	2071	97.73	36	1.7	12	0.57	1967	92.83	140	6.61	12	0.57
		V Unskilled	458	97.03	12	2.54	2	0.42	422	89.41	48	10.17	2	0.42
		.	5306	83.17	80	1.25	994	15.58	5080	79.62	306	4.8	994	15.58
	Own highest education at age 23	Degree/higher	1228	98.63	10	0.8	7	0.56	1195	95.98	43	3.45	7	0.56
		A levels/ diploma	917	98.07	14	1.5	4	0.43	885	94.65	46	4.92	4	0.43
		O levels / GCSE	1134	98.61	15	1.3	1	0.09	1095	95.22	54	4.7	1	0.09
		None	9025	98.02	141	1.53	41	0.45	8641	93.85	525	5.7	41	0.45
		.	4954	82.28	76	1.26	991	16.46	4753	78.94	277	4.6	991	16.46
	Housing tenure at age 23	Own	3535	98.61	40	1.12	10	0.28	3435	95.82	140	3.91	10	0.28
		Rent/other	8409	97.95	134	1.56	42	0.49	8033	93.57	510	5.94	42	0.49
		.	5314	83.19	82	1.28	992	15.53	5101	79.85	295	4.62	992	15.53

**NSHD 1946**		I Professional etc.	254	96.95	6	2.29	2	0.76	250	95.42	10	3.82	2	0.76	235	89.69	25	9.54	2	0.76
		II Intermediate	727	97.19	19	2.54	2	0.27	698	93.32	48	6.42	2	0.27	640	85.56	106	14.17	2	0.27
	Paternal social class at birth	III Non-manual skilled	803	97.45	17	2.06	4	0.49	771	93.57	49	5.95	4	0.49	715	86.77	105	12.74	4	0.49
		III Manual skilled	1374	96.69	43	3.03	4	0.28	1302	91.63	115	8.09	4	0.28	1173	82.55	244	17.17	4	0.28
		IV Partly skilled	903	95.76	34	3.61	6	0.64	844	89.5	93	9.86	6	0.64	753	79.85	184	19.51	6	0.64
		V Unskilled	291	96.36	8	2.65	3	0.99	276	91.39	23	7.62	3	0.99	246	81.46	53	17.55	3	0.99
		.	602	69.84	253	29.35	7	0.81	586	67.98	269	31.21	7	0.81	540	62.65	315	36.54	7	0.81
	Maternal Education at age 6	Continued school	1529	96.65	47	2.97	6	0.38	1461	92.35	115	7.27	6	0.38	1354	85.59	222	14.03	6	0.38
		Left school	2677	97.03	69	2.5	13	0.47	2543	92.17	203	7.36	13	0.47	2279	82.6	467	16.93	13	0.47
		.	748	73.26	264	25.86	9	0.88	723	70.81	289	28.31	9	0.88	669	65.52	343	33.59	9	0.88
		I Professional etc.	240	97.56	4	1.63	2	0.81	227	92.28	17	6.91	2	0.81	214	86.99	30	12.2	2	0.81
		II Intermediate	887	98.89	8	0.89	2	0.22	857	95.54	38	4.24	2	0.22	789	87.96	106	11.82	2	0.22
	Own social class at age 26	III Non-manual skilled	830	98.69	10	1.19	1	0.12	795	94.53	45	5.35	1	0.12	735	87.4	105	12.49	1	0.12
		III Manual skilled	785	98.87	7	0.88	2	0.25	737	92.82	55	6.93	2	0.25	664	83.63	128	16.12	2	0.25
		IV Partly skilled	455	97.85	10	2.15	0	0	419	90.11	46	9.89	0	0	365	78.49	100	21.51	0	0
		V Unskilled	107	97.27	3	2.73	0	0	103	93.64	7	6.36	0	0	96	87.27	14	12.73	0	0
		.	1650	82.13	338	16.82	21	1.05	1589	79.09	399	19.86	21	1.05	1439	71.63	549	27.33	21	1.05
		Degree/higher	401	97.57	8	1.95	2	0.49	385	93.67	24	5.84	2	0.49	357	86.86	52	12.65	2	0.49
	Own highest education at age 26	A levels/ diploma	1019	97.98	17	1.63	4	0.38	988	95	48	4.62	4	0.38	926	89.04	110	10.58	4	0.38
		O levels / GCSE	1192	98.03	21	1.73	3	0.25	1135	93.34	78	6.41	3	0.25	1039	85.44	174	14.31	3	0.25
		None	1709	96.83	47	2.66	9	0.51	1603	90.82	153	8.67	9	0.51	1400	79.32	356	20.17	9	0.51
		.	633	68.06	287	30.86	10	1.08	616	66.24	304	32.69	10	1.08	580	62.37	340	36.56	10	1.08
	Housing tenure at age 26	Own	1685	98.6	19	1.11	5	0.29	1631	95.44	73	4.27	5	0.29	1509	88.3	195	11.41	5	0.29
		Rent/other	1990	97.79	42	2.06	3	0.15	1859	91.35	173	8.5	3	0.15	1664	81.77	368	18.08	3	0.15
		.	1279	79.05	319	19.72	20	1.24	1237	76.45	361	22.31	20	1.24	1129	69.78	469	28.99	20	1.24

Supplementary Table S3. Distribution of socioeconomic indicators in the 1946, 1958, and 1970 cohort

		1970		1958		1946 (weighted)	
		N	*(%)*	*N*	*(%)*	*N*	*(%)*
Paternal social class at birth	I Professional etc.	820	4.29	746	4.02	262	4.89
	II Intermediate	1906	9.98	2133	11.49	748	13.95
	III Non-manual skilled	1924	10.08	1592	8.58	824	15.37
	III Manual skilled	7544	39.51	8376	45.13	1421	26.50
	IV Partly skilled	2473	12.95	1995	10.75	943	17.59
	V Unskilled	1106	5.79	1616	8.71	302	5.63
	.	3320	17.39	2100	11.32	862	16.08
Maternal Education at birth	Continued school	5785	30.30	4337	23.37	1582	29.50
	Left school	11264	59.00	13016	70.14	2759	51.45
	.	2044	10.71	1205	6.49	1021	19.04
Own social class at age 26	I Professional etc.	460	2.41	435	2.34	246	4.59
	II Intermediate	2238	11.72	2136	11.51	897	16.73
	III Non-manual skilled	1830	9.58	4000	21.55	841	15.68
	III Manual skilled	1166	6.11	3016	16.25	794	14.81
	IV Partly skilled	919	4.81	2119	11.42	465	8.67
	V Unskilled	179	0.94	472	2.54	110	2.05
	.	12301	64.43	6380	34.38	2009	37.47
Own highest education at age 26	Degree/higher	2110	11.05	1245	6.71	411	7.67
	A levels/ diploma	894	4.68	935	5.04	1040	19.40
	O levels / GCSE	4908	25.71	1150	6.20	1216	22.68
	None	487	2.55	9207	49.61	1765	32.92
	.	10694	56.01	6021	32.44	930	17.34
Housing tenure at age 26	Own	3809	19.95	3585	19.32	1709	31.87
	Rent/other	4500	23.57	8585	46.26	2035	37.95
	.	10784	56.48	6388	34.42	1618	30.18

Supplementary Table S4. Associations between socioeconomic position in early and adult life and mortality risk: evidence from 3 birth cohort studies, adjusted for sex

		Mortality from 26-43					Mortality from 26-58					Mortality from 26-70			
		HR	*95% CI*		*P*	*P cohort X SEP*	*HR*	*95% CI*		*P*	*P cohort X SEP*	*HR*	*95% CI*		*P*
**BCS 1970**	Paternal social class at birth	1.94	1.20	3.15	0.007										
N=17910	Maternal education at birth	1.32	1.00	1.73	0.048										
	Own social class at age 26	2.36	1.05	2.30	0.038										
	Own education at age 26	1.48	0.68	3.25	0.323										
	Housing tenure at age 26	1.61	1.00	2.60	0.051										
**NCDS 1958**	Paternal social class at birth	1.66	1.03	2.69	0.038		2.42	1.88	3.11	<0.001					
N=17521	Maternal education at birth	1.49	1.08	2.05	0.015		1.34	1.14	1.58	0.001					
	Own social class at age 23	1.58	0.83	3.00	0.156		2.04	1.50	2.79	<0.001					
	Own education at age 23	1.80	0.87	3.70	0.112		1.85	1.23	2.78	0.004					
	Housing tenure at age 23	1.30	0.87	1.94	0.196		1.47	1.23	1.76	<0.001					
**NSHD 1946**	Paternal social class at age 4	2.74	1.02	7.32	0.045	0.879	2.38	1.49	3.83	<0.001	0.776	1.89	1.37	2.60	<0.001
N=5353	Maternal education at age 6	1.10	0.61	1.98	0.759	0.584	1.09	0.81	1.47	0.551	0.441	1.27	1.05	1.55	0.015
	Own social class at age 26	5.07	0.86	29.97	0.072	0.970	3.05	1.60	5.84	<0.001	0.531	2.36	1.60	3.48	<0.001
	Own education at age 26	3.37	0.94	12.07	0.062	0.921	2.24	1.33	3.77	0.003	0.912	2.64	1.88	3.71	<0.001
	Housing tenure at age 26	2.06	1.03	4.12	0.040	0.856	1.97	1.44	2.70	<0.001	0.445	1.68	1.39	2.03	<0.001

Adjusted for sex

Cox regression with ridit scores, Hazard Ratios, 95% CI

Supplementary Table S5. Associations between socioeconomic position in early and adult life and mortality risk: evidence from 3 birth cohort studies, mutually adjusted for multiple indicators of socioeconomic position

a)

			**Mortality from 26-43**				**Mortality from 26-58**				**Mortality from 26-70**			
		N	*HR*	*95% CI*		*P*	*HR*	*95% CI*		*P*	*HR*	*95% CI*		*P*
**BCS 1970**	Paternal social class at birth [a]	17910	1.54	0.85	2.78	0.154								
	Own social class at age 26 [a]	17910	2.53	0.54	11.88	0.215								
	Parental + own social class [b]	17910	2.15	1.13	2.73	0.023								
**NCDS 1958**	Paternal social class at birth [a]	17521	1.44	0.83	2.51	0.192	2.00	1.54	2.60	<0.001				
	Own social class at age 23 [a]	17521	1.67	0.83	3.38	0.144	1.81	1.33	2.46	<0.001				
	Parental + own social class [b]	17521	1.62	0.93	2.85	0.097	2.23	1.69	2.95	<0.001				
**NSHD 1946**	Paternal social class at age 4 [a]	5353	1.75	0.60	5.11	0.304	1.76	1.02	3.04	0.043	1.48	1.03	2.13	0.035
	Own social class at age 26 [a]	5353	5.21	1.30	20.96	0.020	2.59	1.27	5.30	0.010	2.10	1.37	3.23	0.001
	Parental + own social class [b]	5353	3.91	0.94	18.65	0.059	2.72	1.55	4.84	<0.001	2.13	1.49	3.04	<0.001

[a] *Mutually adjusted for sex, paternal social class, and adult social class. Cox regression with ridit scores, Hazard Ratios, 95% CI*

[b] *Parental + own SEP indicates composite score combining sex only adjusted paternal social class with own social class*

b)

		Mortality from 26-43					Mortality from 26-58				Mortality from 26-70			
		N	*HR*	*95% CI*		*P*	*HR*	*95% CI*		*P*	*HR*	*95% CI*		*P*
**BCS 1970**	Paternal social class at birth [a]	17910	1.50	0.83	2.71	0.174								
	Own social class at age 26 [a]	17910	2.40	0.52	11.13	0.236								
	Housing tenure at age 26 [a]	17910	1.61	1.09	2.40	0.020								
	Parental + own SEP [b]	17910	1.59	1.04	2.44	0.022								
**NCDS 1958**	Paternal social class at birth [a]	17521	1.44	0.83	2.50	0.191	1.99	1.53	2.60	<0.001				
	Own social class at age 23 [a]	17521	1.59	0.79	3.22	0.188	1.70	1.26	2.30	0.001				
	Housing tenure at age 23 [a]	17521	1.38	0.92	2.07	0.116	1.43	1.20	1.71	<0.001				
	Parental + own SEP [b]	17521	1.51	0.91	2.54	0.130	1.98	1.54	2.55	<0.001				
**NSHD 1946**	Paternal social class at age 4 [a]	5353	1.60	0.55	4.65	0.387	1.62	0.94	2.78	0.079	1.40	0.98		
	Own social class at age 26 [a]	5353	4.50	1.14	17.79	0.032	2.26	1.10	4.66	0.028	1.90	1.22		
	Housing tenure at age 26 [a]	5353	1.82	0.94	3.49	0.074	1.80	1.32	2.46	<0.001	1.57	1.30		
	Parental + own SEP [b]	5353	3.29	0.97	13.80	0.052	2.47	1.51	4.12	<0.001	1.98	1.45	2.70	<0.001

[a] *Mutually adjusted for sex, paternal social class, adult social class, and adult housing tenure. Cox regression with ridit scores, Hazard Ratios, 95% CI*
[b] *Parental + own SEP indicates composite score combining sex only adjusted paternal social class with own social class and housing*

Supplementary Table S6. Associations between socioeconomic position in early and adult life and mortality risk: evidence from 3 birth cohort studies, estimated on the absolute scale

			**Mortality from 26-43**				**Mortality from 26-58**				**Mortality from 26-70**			
		N	*RD*	*95% CI*		*P*	*RD*	*95% CI*		*P*	*RD*	*95% CI*		*P*
**BCS 1970**	Paternal social class at birth	17910	-0.18%	-0.31%	-0.05%	0.007								
	Maternal education at birth	17910	-0.41%	-0.81%	0.00%	0.051								
	Own social class at age 26	17910	0.02%	-0.15%	0.19%	0.807								
	Own education at age 26	17910	0.09%	-0.13%	0.30%	0.427								
	Housing tenure at age 26	17910	-0.70%	-1.41%	0.02%	0.056								
**NCDS 1958**	Paternal social class at birth	17521	-0.19%	-0.35%	-0.04%	0.017	-1.08%	-1.38%	-0.78%	<0.001				
	Maternal education at birth	17521	-0.56%	-1.04%	-0.09%	0.020	-1.52%	-2.38%	-0.66%	0.001				
	Own social class at age 23	17521	-0.19%	-0.40%	0.01%	0.068	-0.95%	-1.33%	-0.56%	<0.001				
	Own education at age 23	17521	-0.23%	-0.48%	0.02%	0.076	-0.79%	-1.28%	-0.30%	0.002				
	Housing tenure at age 23	17521	-0.37%	-0.93%	0.18%	0.185	-2.01%	-2.94%	-1.09%	<0.001				
**NSHD 1946**	Paternal social class at age 4	5353	-0.27%	-0.71%	0.17%	0.224	-1.06%	-1.74%	-0.37%	0.003	-1.86%	-2.87%	-0.84%	<0.001
	Maternal education at age 6	5353	0.43%	-0.67%	1.53%	0.445	0.30%	-1.72%	2.41%	0.865	-2.94%	-5.72%	-0.16%	<0.001
	Own social class at age 26	5353	-0.55%	-1.10%	0.00%	0.049	-1.45%	-2.32%	-0.58%	0.002	-2.61%	-3.76%	-1.46%	<0.001
	Own education at age 26	5353	-0.71%	-1.37%	-0.05%	0.036	-1.57%	-2.56%	-0.59%	0.002	-4.04%	-5.45%	-2.62%	<0.001
	Housing tenure at age 26	5353	-1.25%	-2.36%	-0.14%	0.027	-4.28%	-6.24%	-2.31%	<0.001	-7.31%	-9.99%	-4.64%	<0.001

Adjusted for sex
Logistic regression, Risk difference between advantaged and disadvantaged SEP 95% CI

Supplementary Table S7. Socioeconomic position in early and adult life and mortality risk: evidence from 3 birth cohort studies, stratified by sex

				Mortality from 26-43				Mortality from 26-58				Mortality from 26-70			
			N	*HR*	*95% CI*		*P*	*HR*	*95% CI*		*P*	*HR*	*95% CI*		*P*
**Males**	**BCS 1970**	Paternal social class at birth	9239	2.45	1.39	4.30	0.002								
		Maternal education at birth	9239	1.56	1.11	2.19	0.010								
		Own social class at age 26	9239	2.52	0.65	9.77	0.166								
		Own education at age 26	9239	1.94	0.66	5.75	0.216								
		Housing tenure at age 26	9239	1.57	0.99	2.49	0.053								
	**NCDS 1958**	Paternal social class at birth	9007	1.58	0.85	2.93	0.148	2.44	1.76	3.38	<0.001				
		Maternal education at birth	9007	1.49	1.00	2.22	0.049	1.30	1.05	1.60	0.017				
		Own social class at age 23	9007	1.47	0.66	3.27	0.335	2.00	1.38	2.90	<0.001				
		Own education at age 23	9007	1.96	0.74	5.17	0.172	1.71	1.03	2.83	0.037				
		Housing tenure at age 23	9007	1.24	0.73	2.12	0.418	1.31	1.01	1.70	0.046				
	**NSHD 1946**	Paternal social class at age 4	2808	2.69	0.69	10.44	0.152	2.08	1.09	3.98	0.027	1.51	0.99	2.32	0.057
		Maternal education at age 6	2808	0.67	0.31	1.45	0.305	0.83	0.57	1.21	0.321	1.08	0.85	1.39	0.525
		Own social class at age 26	2808	5.61	0.58	53.94	0.133	3.11	1.48	6.51	0.003	2.25	1.47	3.46	<0.001
		Own education at age 26	2808	4.21	0.87	20.32	0.073	1.97	1.04	3.77	0.039	2.07	1.37	3.12	0.001
		Housing tenure at age 26	2808	2.32	0.89	6.01	0.084	1.94	1.24	3.05	0.004	1.64	1.27	2.13	<0.001
**Females**	**BCS 1970**	Paternal social class at birth	8671	1.21	0.53	2.78	0.648								
		Maternal education at birth	8671	1.01	0.63	1.62	0.951								
		Own social class at age 26	8671	3.67	0.51	26.59	0.182								
		Own education at age 26	8671	1.39	0.38	5.04	0.606								
		Housing tenure at age 26	8671	1.91	1.13	3.23	0.016								
	**NCDS 1958**	Paternal social class at birth	8514	1.84	0.83	4.07	0.135	2.21	1.47	3.31	<0.001				
		Maternal education at birth	8514	1.47	0.87	2.50	0.148	1.38	1.06	1.79	0.016				
		Own social class at age 23	8514	1.83	0.81	4.13	0.146	1.90	1.23	2.94	0.004				
		Own education at age 23	8514	1.56	0.58	4.20	0.381	1.89	1.07	3.34	0.027				
		Housing tenure at age 23	8514	1.39	0.86	2.24	0.181	1.54	1.20	1.98	0.001				
	**NSHD 1946**	Paternal social class at age 4	2545	2.80	0.62	12.65	0.181	2.84	1.45	5.55	0.002	2.65	1.66	4.24	<0.001
		Maternal education at age 6	2545	2.32	0.88	6.10	0.087	1.69	1.04	2.74	0.034	1.68	1.22	2.32	0.001
		Own social class at age 26	2545	4.64	0.50	43.41	0.172	2.99	1.06	8.47	0.040	2.53	1.27	5.07	0.010
		Own education at age 26	2545	2.53	0.33	19.28	0.364	2.78	1.13	6.83	0.026	4.45	2.46	8.06	<0.001
		Housing tenure at age 26	2545	1.86	0.73	4.77	0.193	2.01	1.25	3.24	0.004	1.73	1.29	2.34	<0.001

Cox regression with ridit scores, Hazard Ratios, 95% CI

Supplementary Table S8. Associations between socioeconomic position in early and adult life and mortality risk: evidence from 3 birth cohort studies, stratified by sex and shown on the absolute scale

				Mortality from 26-43				Mortality from 26-58				Mortality from 26-70			
			N	*RD*	*95% CI*		*P*	*RD*	*95% CI*		*P*	*RD*	*95% CI*		*P*
**Males**	**BCS 1970**	Paternal social class at birth	9239	-0.46%	-0.73%	-0.19%	0.001								
		Maternal education at birth	9239	-0.91%	-1.59%	-0.23%	0.009								
		Own social class at age 26	9239	-0.37%	-1.01%	0.26%	0.227								
		Own education at age 26	9239	-0.36%	-0.95%	0.23%	0.220								
		Housing tenure at age 26	9239	-0.89%	-1.79%	0.02%	0.066								
	**NCDS 1958**	Paternal social class at birth	9003	-0.25%	-0.51%	0.01%	0.057	-1.29%	-1.74%	-0.83%	<0.001				
		Maternal education at birth	9003	-0.77%	-1.52%	-0.02%	0.043	-1.62%	-2.93%	-0.31%	0.015				
		Own social class at age 23	9003	-0.21%	-0.55%	0.12%	0.201	-1.07%	-1.62%	-0.53%	<0.001				
		Own education at age 23	9003	-0.34%	-0.75%	0.08%	0.109	-0.88%	-1.56%	-0.19%	0.013				
		Housing tenure at age 23	9003	-0.39%	-1.36%	0.58%	0.424	-1.68%	-3.23%	-0.13%	0.034				
	**NSHD 1946**	Paternal social class at age 4	2620	-0.50%	-1.09%	0.10%	0.100	-1.12%	-2.10%	-0.13%	0.026	-1.44%	-2.95%	0.08%	0.063
		Maternal education at age 6	2620	1.50%	-0.05%	3.05%	0.058	2.11%	-0.51%	4.72%	0.004	-0.56%	-4.57%	3.45%	0.783
		Own social class at age 26	2620	-0.69%	-1.46%	0.07%	0.076	-1.65%	-2.75%	-0.56%	0.003	-2.90%	-4.43%	-1.38%	<0.001
		Own education at age 26	2620	-0.75%	-1.60%	0.09%	0.080	-1.34%	-2.62%	-0.06%	0.040	-3.31%	-5.20%	-1.43%	0.001
		Housing tenure at age 26	2620	-1.46%	-3.02%	0.10%	0.066	-4.48%	-7.48%	-1.49%	0.004	-7.95%	-12.09%	-3.80%	<0.001
**Females**	**BCS 1970**	Paternal social class at birth	8,670	-0.02%	-0.20%	0.16%	0.802								
		Maternal education at birth	8,670	0.03%	-0.42%	0.47%	0.091								
		Own social class at age 26	8,670	-0.25%	-0.65%	0.15%	0.203								
		Own education at age 26	8,670	-0.10%	-0.43%	0.23%	0.555								
		Housing tenure at age 26	8,670	-0.62%	-1.15%	-0.10%	0.020								
	**NCDS 1958**	Paternal social class at birth	8,510	-0.13%	-0.32%	0.06%	0.172	-0.81%	-1.21%	-0.41%	<0.001				
		Maternal education at birth	8,510	-0.34%	-0.89%	0.21%	0.220	-1.33%	-2.46%	-0.20%	0.021				
		Own social class at age 23	8,510	-0.17%	-0.38%	0.03%	0.101	-0.72%	-1.17%	-0.27%	0.002				
		Own education at age 23	8,510	-0.11%	-0.37%	0.14%	0.392	-0.63%	-1.21%	-0.04%	0.035				
		Housing tenure at age 23	8,510	-0.34%	-0.83%	0.16%	0.184	-1.90%	-3.01%	-0.79%	0.001				
	**NSHD 1946**	Paternal social class at age 4	2405	-0.04%	-0.69%	0.62%	0.915	-0.99%	-1.94%	-0.03%	0.043	-2.32%	-3.66%	-0.99%	0.001
		Maternal education at age 6	2405	-1.01%	-2.81%	0.79%	0.273	-2.76%	-5.68%	0.15%	0.063	-6.00%	-9.94%	-2.06%	0.003
		Own social class at age 26	2405	-0.42%	-1.10%	0.27%	0.226	-1.23%	-2.48%	0.01%	0.053	-2.29%	-3.99%	-0.60%	0.009
		Own education at age 26	2405	-0.67%	-1.73%	0.40%	0.218	-2.03%	-3.65%	-0.41%	0.014	-5.66%	-7.89%	-3.43%	<0.001
		Housing tenure at age 26	2405	-1.07%	-2.57%	0.43%	0.162	-4.06%	-6.83%	-1.28%	0.004	-6.61%	-10.22%	-3.00%	<0.001

Logistic regression, Risk difference between advantaged and disadvantaged SEP 95% CI
